# Featherweight long read alignment using partitioned reference indexes

**DOI:** 10.1038/s41598-019-40739-8

**Published:** 2019-03-13

**Authors:** Hasindu Gamaarachchi, Sri Parameswaran, Martin A. Smith

**Affiliations:** 1 Kinghorn Centre for Clinical Genomics, Garvan Institute of Medical Research, 370 Victoria St, Darlinghurst, NSW Australia; 2 School of Computer Science and Engineering, UNSW Sydney, Kensington, NSW Australia; 3 St-Vincent’s Clinical School, Faculty of Medicine, UNSW Sydney, Darlinghurst, NSW Australia

## Abstract

The advent of Nanopore sequencing has realised portable genomic research and applications. However, state of the art long read aligners and large reference genomes are not compatible with most mobile computing devices due to their high memory requirements. We show how memory requirements can be reduced through parameter optimisation and reference genome partitioning, but highlight the associated limitations and caveats of these approaches. We then demonstrate how these issues can be overcome through an appropriate merging technique. We incorporated multi-index merging into the Minimap2 aligner and demonstrate that long read alignment to the human genome can be performed on a system with 2 GB RAM with negligible impact on accuracy.

## Introduction

Long read sequencing has revolutionised genome research by facilitating the characterisation of large structural variations, repetitive regions, and de-novo assembly of whole genomes. Pacific Biosciences (PacBio) and Oxford Nanopore Technologies (ONT) are leading manufacturers that produce long read sequencers. In particular, ONT manufacture sequencers smaller than the size of a mobile phone that can nevertheless output more than 1 TB of data in 48 hours. Such highly portable sequencers have realised the possibility of performing genome sequencing in the field. For instance, ONT’s MinION sequencer has been used for Ebola virus surveillance in New Guinea^1^, mobile Zika virus surveillance in Brazil^2^, and for experiments on the International space station^3^.

The advent of highly portable DNA sequencers raises the need for local data processing on devices such as mobile phones, tablets and laptops. Facilitating genomic data analysis on mobile devices avoids the need for high speed internet connections and enables real-time genomic tests and experiments. For Nanopore sequencers, a pico-ampere ionic current signal is produced for each DNA read, which is subsequently converted to nucleotide bases via applied machine learning models. Until recently, a high performance workstation (Quad-core i7 or Xeon processor, 16 GB RAM, 1 TB SSD) was required for live base calling, the process of converting ionic signal to nucleotide sequences.

Most genomic analyses depend on base calling as an initial step, which can be efficiently performed through GPGPU software implementations on graphics cards or, quite conveniently, on dedicated portable hardware (ONT manufacture one such device, termed ‘MinIT’). Next, base called reads are typically aligned/mapped to a reference, in the case of reference guided assembly, or aligned to themselves in the case of de-novo assembly. Subsequent analyses (i.e. consensus sequence generation, variant calling, methylation detection, etc) should follow this alignment step. Therefore, an alignment tool that can run on portable devices such as mobile phones, tablets and laptops is the next step in realising the full portability of a complete Nanopore processing pipeline.

Minimap2^4^ is a general purpose mapper/aligner that is compatible with both DNA and RNA sequences. Minimap2 can align both long reads and short reads, either to a reference or an assembly contig. Minimap2 first employs hashing followed by chaining for coarse-grained alignment. Then it performs an optional base level alignment using an optimised implementation of the Suzuki-Kasahara Dynamic Programming (DP) formulation^5^. Minimap2 stands out as the current aligner of choice for long reads, among other long read aligners such as BLASR^6^, GraphMap^7^, Kart^8^, NGMLR^9^ and LAMSA^10^; not only is it 30 times faster than existing long read aligners, but its accuracy is on par or superior to other algorithms^4^. The hash table based approach in Minimap2 has been shown to be effective for long reads. In contrast, FM-index^11^ based short read aligners such as BWA^12^ and Bowtie^13^ have been shown to fail with ultra long reads (i.e. several hundred kilobases or more)^14^.

Most alignment tools build an index of reference sequences that is stored in volatile memory. Whilst this is manageable for small genomes such as individual bacteria (~5 Mb), fungi (~50 Mb) or insects (~400 Mb), most vertebrates and some plant species require large amounts of memory, as their genomes are in the 1–100 Gb range. For example, building an index for the GRCh38 human genome reference requires over 11.2 GB of volatile memory, and at the very least 8.8 GB to map nanopore reads to a pre-computed index with Minimap2.

To accelerate the development and uptake of real-time genomic applications in field research and point of care medical testing, long read sequence alignment should be performed on ultra-portable computing devices. These can include mobile phones, microcomputer boards, Field-Programmable Gate Arrays (FPGAs), and other embedded systems, such as ONT’s “MinIT” and “Mk1c MinION” devices. Such hardware rarely have more than 4 GB of RAM, therefore more expensive and less portable equipment is typically required for sequence alignment (high-end laptops, internet connectivity, power generation, etc).

Here, we describe strategies for long read alignment to large reference genomes (or collections of genomes) using low amounts of memory. We present an efficient approach to achieve this by splitting a genome index into smaller partitions. Although partitioned indexes are not a novel concept^15–17^, we expose the caveats of their use on the accuracy of entailing alignments. We present a solution to these issues by merging multi-part alignments via serialisation of internal data structures of the Minimap2 aligner, and demonstrate how this strategy produces alignments that are almost indiscernible from a classical single index using simulated long reads, Nanopore NA12878 reference human genome sequencing data, and a 470 kb long chromothriptic read from a human cancer cell line.

## Results

### Effect of parameters on memory usage

With default options, Minimap2 requires more than 11 GB of memory to create an index from the human reference genome sequence and align Nanopore reads against it (Supplementary Table S1). Although the pre-calculated index can be saved to disk, 7.7 GB are nonetheless required to subsequently load the index into memory, and between 8.8 and 11.3 GB are required when intermediate data structures during alignment are included. This exceeds the average RAM capacities of high-end mobile phones and mid-range laptops. Hence, running Minimap2 on human data with default options on a typical laptop with 8 GB memory or a typical mobile phone with 2 GB of memory is not feasible.

We therefore tested the relative effect of alignment parameters on peak memory usage in Minimap2 (see Materials and methods) to investigate if parameter optimisation alone can significantly reduce the memory requirements without compromising alignment quality. For this purpose, we used Sequins—synthetic DNA spike-in controls that are designed from the reverse or ‘mirrored’ human genome sequence^18^. This chirality reproduces diverse properties of the human genome, such as nucleotide frequencies, complexity, repetitiveness, somatic variation, etc. As detailed in Materials and methods, we aligned Nanopore sequencing data from Sequins to both native and reversed (not complemented) human reference genomes to compare the relative impact of Minimap2 parameters on alignment accuracy. Specifically:

- *k* the minimiser k-mer length (default = 15 for ONT data);
- *w* the minimiser window size (default = 10);
- *t* the number of threads (default = 4);
- *K* the number of query bases loaded into memory at a time (default = 500 M).

**Figure 1:**
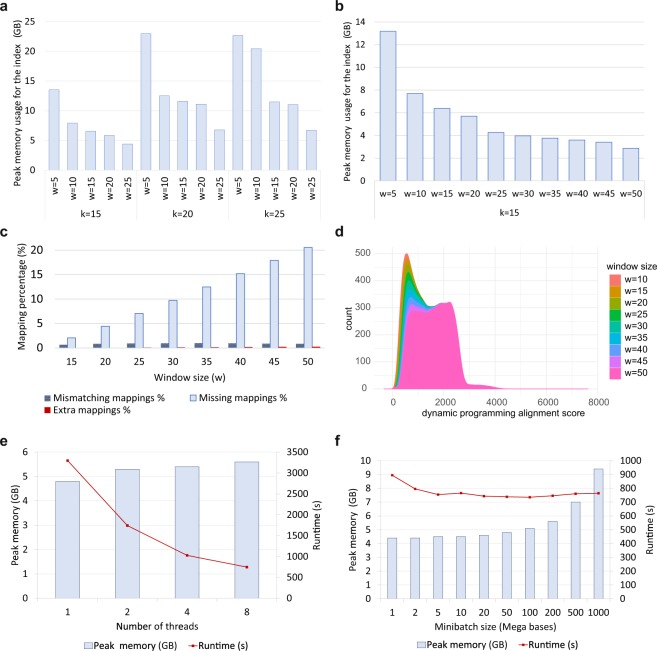
Effect of parameters on memory usage, performance and accuracy. (**a**) Peak memory usage of the index for different combinations of *k* and *w*. (**b**) Peak memory usage of the index for a large range of *w* with *k* = 15. (**c**) The effect of *w* on sensitivity and error relative to the default window size. The x-axis is the minimiser window size (*w*). The k-mer size is held constant at 15 for all values of *w*. The y-axis shows the number of missing mappings/mismatches or extra mapping (compared to the mappings from default *w* = 10) as a percentage of the number of reads. (**d**) Distribution of the dynamic programming alignment score for different minimiser window sizes (*w*). The x-axis is the score and the y-axis is the smoothed number of mappings for a particular score. Note that the distribution is smoothed to show the trend. (**e**) Effect of the number of threads on memory and performance. The parameters *k*, *w* and *K* were held constant at 15, 25 and 200 M respectively while changing the number of threads. Both the peak memory usage and the runtime were measured on a PC with an Intel i7-6700 CPU and 16 GB of RAM. (**f**) Effect of the number of query bases loaded at a time. The parameters *k*, *w* and *t* were held constant at 15, 25 and 8 respectively.

Parameters *k* and *w* considerably affect the peak memory usage for holding the index in memory (Fig. 1a). For an index without homo-polymer compression, *k* = 15 consumed the least amount of memory out of the values tested, as expected. In fact, the default k-mer size for ONT data in Minimap2 (pre-set command line parameter *map-ont*) is 15.

Unsurprisingly, parameter *w* has the most prominent impact on memory usage, which decreases considerably when increasing *w* (Fig. 1b). At *w* = 50, memory usage is capped at 3 GB, but the sensitivity (see Materials and methods) is substantially reduced compared to the default value of parameter *w* (missing mappings in Fig. 1c). A larger *w* of 50 reduces sensitivity compared to the default value of *w* by 20%, whereas a *w* of 25 entails an apparent reduction in sensitivity of about 7% while nonetheless requiring more than 4 GB of memory. Although sufficient for a computer with 8 GB of RAM, this is still too high for smaller devices.

Importantly, the amount of mismatched mappings in mapped reads are not significantly affected by the *w* parameter (mismatches in Fig. 1c), nor are the high-quality alignments as demonstrated by their dynamic programming (DP) alignment score distributions (cf. DP score > 2000 in Fig. 1d). However, lower (≈1000) DP scores are less frequent with increasing window size, as are alignments with high MAPQ scores, while those with low MAPQ scores are more prevalent (Supplementary Fig. S1). The effect of the window size is also apparent with simulated PacBio reads, where both the sensitivity and error-rate of alignments are negatively affected (Supplementary Fig. S2).

The number of threads marginally increases peak memory usage (Fig. 1e). Only about 0.8 GB of additional memory was consumed when moving from 1 to 8 threads, while producing a 6-fold gain in speed. Hence, reducing the number of threads for the sake of reduced memory usage is not an efficient solution.

Intuitively, the number of query bases loaded to the memory at once (also known as the mini-batch size) heavily impacts peak memory usage. This affects the size of the internal data structures used for mapping, but does not affect the index size. Hence, this parameter does not affect alignment accuracy or the sensitivity. A lower mini-batch size reduces the peak memory usage, at the cost of reduced multi-threading efficiency (Fig. 1f). The runtime drops when changing the mini-batch size from 1 M to 5 M. However, the runtime is relatively stable from a mini-batch size of 5 M onwards. It is important to note that the values in Fig. 1f are only valid for 8 threads. A large number of threads would require a large mini-batch size for optimal performance.

Although parameter adjustments (small minimiser window size value and mini-batch sizes from 5 M–20 M in particular) can be suitable for systems with limited RAM (for 8 CPU threads or less), tuning parameters alone cannot bring down the memory usage to a value lesser than 4 GB due to a substantial loss of sensitivity. As a consequence, this inspired us to investigate the use and suitability of partitioning (or splitting) the reference sequences into distinct indexes.

### Caveats of naive partitioned indexes

**Figure 2:**
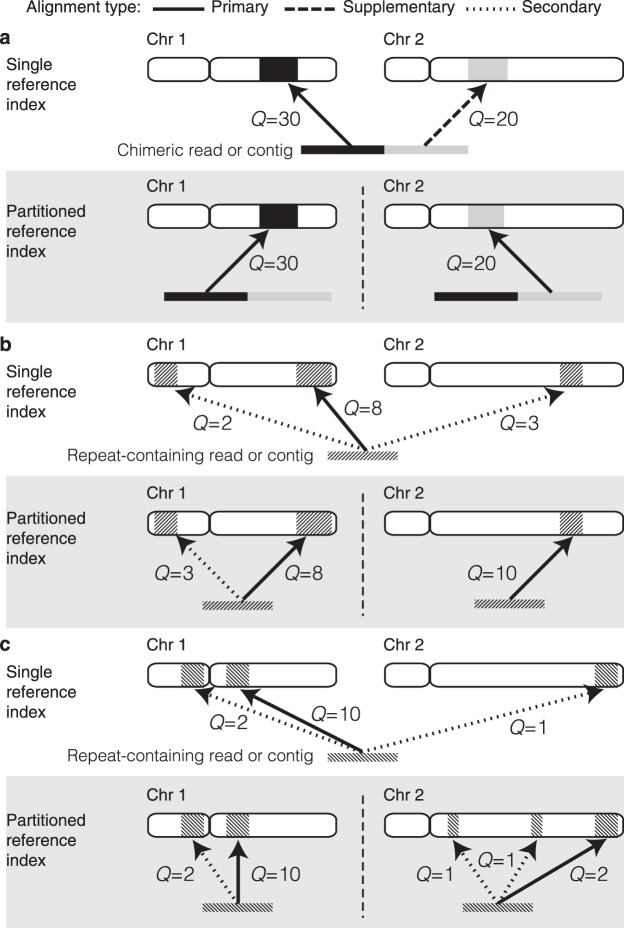
Effect of aligning sequences to single vs partitioned indexes. Uniquely mapping chimeric reads (**a**) can be reconstructed from a partitioned reference index with relative ease. However, sequences (or sub-sequences) that are difficult to map (i.e. low complexity regions, repetitive elements, etc) can cause artefacts when aligning to a partitioned reference index. (**b**) An example where one partition (chr2) contains less homologous sequences to the query sequence, producing the situation where the best alignment when using a single reference is not achieved. (**c**) An example where a partitioned reference introduces several additional low quality mappings that would be dismissed with a single reference index. *Q*: mapping quality score.

Minimap2 allows the reference index to be split by a user-specified number of bases through the option *I*, effectively dividing a reference into smaller indexes of comparable size. This facilitates parallel computation and, in theory, enables lower peak memory requirements. However, this feature is not ideal for mapping single reads to large references, mainly because global contiguous information about the reference is unavailable. As a result, several mapping artefacts can occur, as listed below and in Fig. 2 (N.B. these may not be as prominent when overlapping reads–the application for which index partitioning in Minimap2 was originally developed).

1. The mapping quality is incorrect.
  The mapping quality estimated in Minimap2 is accurate as it deliberately lowers the mapping quality for repetitive hits. However, this is not possible when only a fraction of a whole genome is present in the index (see Supplementary Materials of Li, H.^4^). In a partitioned index, if the same repeat lies across different partitions, the mapping quality will be overestimated (Fig. 2b).
2. Incorrect alignment flags.
  For a chimeric read where different sub-sequences map to different chromosomes, the supplementary mappings would be marked as primary mappings (Fig. 2a). A repeat-containing read that maps to multiple locations across different partitions will have multiple primary alignments instead of a single primary alignment (Fig. 2b,c).
3. Large output files.
  A spurious unmapped record will be printed for each partition of the index where a particular read does not map to. Furthermore, if a maximal amount of secondary alignments are specified, that number of secondary alignments would be output for each partition (Fig. 2c). Hence, the more partitions used, the larger the output files will be. Such large outputs not only waste disk space, but they are also time consuming to parse or sort.
4. Multiple hits of the same query may not be adjacent in the output^19^.
  This causes difficulties to analyse or evaluate mapping results. For instance, the *Mapeval* utility in *Paftools* (a tool bundled with Minimap2 for evaluating alignment accuracy) is not compatible with such outputs. Sorting by the read identifier would fix the issue, but requires significant computation for large files.
5. Incomplete headers in the sequence alignment/map (SAM) output.

  For a partitioned index, Minimap2 suppresses the reference sequence dictionary (SQ lines) in the SAM header. Users must manually add SQ lines to the header for compatibility with downstream analysis tools.

We resolved these issues by serialising and storing the internal state of Minimap2 while mapping reads, then merging the output and processing the result *a posteriori* (see Materials and methods). The accuracy of this technique is discussed below.

### Effect of using a partitioned index on alignment accuracy

We compared the alignment accuracy between a single reference index and a partitioned index, with and without merging the output. The following acronyms will be used in the subsequent text (see Materials and methods for more details):

- *single-idx*: Aligning reads to a single reference index;
- *part-idx-no-merge*: Aligning reads to a partitioned index without merging the output;
- *part-idx-merged*: Aligning reads to a partitioned index while applying our merging technique.

#### Synthetic long reads

**Figure 3:**
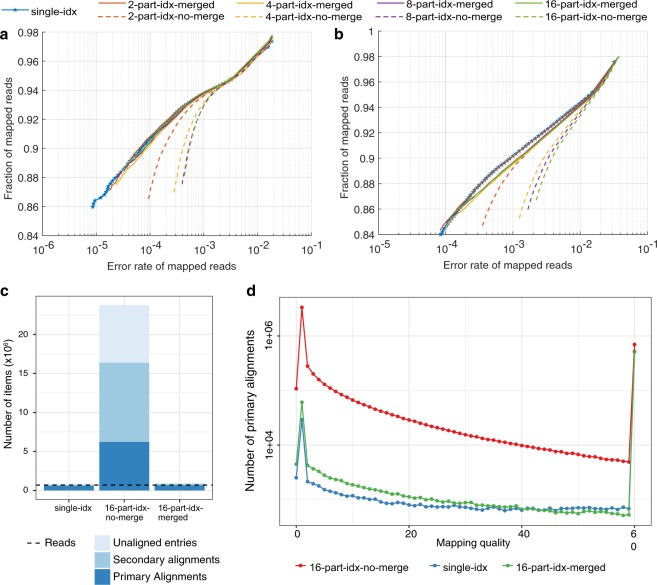
Effect of using partitioned indexes versus a single reference index on alignment quality. (**a**) Base-level and (**b**) locus- or block-level alignment accuracy from synthetic long reads. The x-axis shows the error rate of alignments in log scale (see Materials and methods). The y-axis shows the fraction of aligned reads out of all input reads. Each point in the plot corresponds to a mapping quality threshold that varies from 0 (top right) to 60 (bottom left). These plots are akin to precision-recall plots with the x-axis inverted. (**c** and **d**) Alignment statistics for Nanopore whole genome sequencing data from NA12878^14^ using a 16-part index. (**c**) The number of total entries (primary + secondary + unaligned) for *single-idx*, *16-part-idx-merged*, and *16-part-idx-no-merge*, in log scale. The dotted horizontal line represents the number of reads. (**d**) Number of primary mappings in function of Minimap2 mapping quality (log scale).

Synthetic long reads were used as a ground truth for the evaluation of alignment accuracy (see Materials and methods). The accuracy of *part-idx-merged* is similar to *single-idx*, despite employing significantly more partitions (Fig. 3a,b) as exemplified by the overlap of their respective curves. In contrast, the results of *part-idx-no-merge* are considerably less accurate, in particular for larger quantities of index partitions. A lower error rate is observed for *part-idx-merged* when compared to *single-idx* for the lowest mapping quality values, but this effect is marginal and is associated with low sensitivity.

#### For real Nanopore NA12878 reads

As no ground truth is available for biological data, we evaluated alignment accuracy by comparing the number of primary/secondary alignments and unmapped reads across single and multi-partition indexes. When using *single-idx*, Minimap2 outputs 12.1 GB of base-level alignment data in SAM format, whereas *part-idx-no-merge* generates much larger output (180 GB). However, *part-idx-merged* generates 12.4 GB of data–proportional to the output produced with *single-idx*. Hence, *part-idx-merged* reduces disk usage by about 14-fold compared to *part-idx-no-merge*. Peak disk usage is also minimised in *part-idx-merged* as only intermediate alignments are serialised as temporary binary files. The resulting size of temporary files generated with *part-idx-merged* is 29.2 GB, thus achieving maximal disk usage of 41.6 GB, 4 times less than *part-idx-no-merge*. The increased output produced by *part-idx-no-merge* is due to redundant unmapped entries and spurious mappings (Fig. 3c and Supplementary Table S2).

The number of total entries (primary + secondary + unaligned) for *single-idx* and *part-idx-merged* are comparable to the number of input reads (689,781), while *part-idx-no-merge* generates abundant—presumably spurious—hits. Furthermore, the distribution of mapping qualities for primary alignments between *part-idx-merged* and *single-idx* are quite similar (Fig. 3d). Interestingly, *part-idx-merged* produces slightly more primary alignments with lower mapping quality scores than *single-idx*, a likely consequence of sampling less repetitive regions in partitioned indexes. All the strategies produce almost the same amount of mappings with quality = 60. In contrast, *part-idx-no-merge* has a very high number of spurious mappings for mapping qualities between 0 to 59.

A more detailed comparison of 689,781 ONT reads aligned using *single-idx* and *16-part-idx-merged* revealed the following: 120,623 (17.49%) reads were unmapped in both; 152 (0.02%) reads mapped only in *single-idx*; 6,554 (0.95%) reads mapped only in *16-part-idx-merged*; 562,452 (81.54%) reads mapped in both. Less than 1% of all reads presented discordant mappings when using a single or a multi-part index. Of those discordant mappings, 6,423 (95.8%) overlapped regions in the human genome annotative as repeat elements or low-complexity sequences, the majority of which were satellites and ALR/Aplha repeats from centromeres (Supplementary Figs S3 and S4). Furthermore, most (97.7%) of these index-specific unique alignments stem from the multi-part index, which suggests that a reduced search space can help Minimap2 map less complex sequences, presumably through more frequent recourse of the dynamic programming step in Minimap2.

Among the 562,452 reads that mapped in both *single-idx* and *16-part-idx-merged*, 545,306 (96.95%) had the exact same primary mappings (same chromosome, strand and position). Out of the remaining 17,146 aligned reads (3.05%): 2,748 (16.02%) of mapping coordinates overlapped by at least 10% in both sets; 952 (5.55%) were classified as supplementary mappings in *16-part-idx-merged* and as primary mappings in *single-idx*; 3,891 (22.69%) were classified as secondary mappings in *16-part-idx-merged* and as primary mappings in *single-idx*.

Of the 17,146 reads with disparate mappings, 50.5% had higher DP alignment scores for the single index, while 42.9% had higher scores in the 16-part index (Pearson’s correlation = 0.93, Supplementary Figs S5 and S6). This effect was similarly observed for MAPQ scores, with 15.2% and 12.8%, respectively, suggesting that alignments are of marginally better quality when generated with a single index. Again, these disparate mappings are largely composed of repetitive and viral sequences (Supplementary Fig. S7). When the 2,748 overlapped reads were removed from the 17,146 disparate mappings, the trend of DP scores, MAPQs, and repeat distributions are very similar to when those are not removed (Supplementary Fig. S8).

#### For an ultra-long chromothriptic read

**Figure 4:**
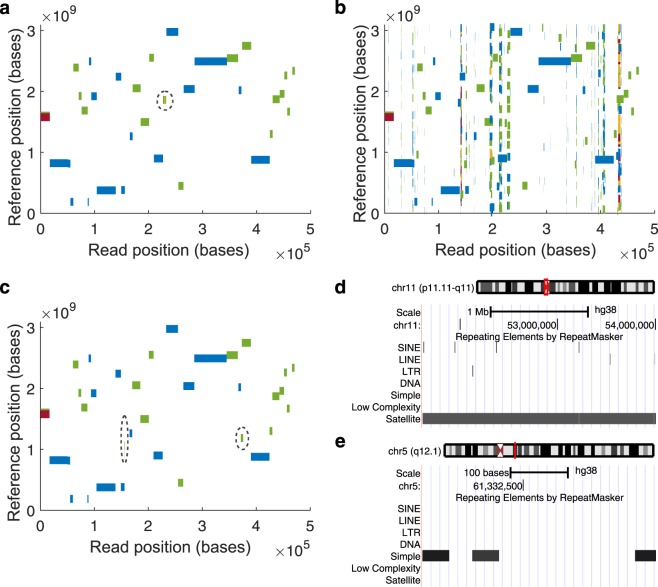
Alignment of an ultra-long ONT read from a chromothriptic region. Mapping coordinates in the entire human reference genome (y-axis) in function of the position in the read, showing where sub-sequences of the chimeric read map in the genome for (**a**) *single-idx*, (**b**) *part-idx-no-merge*, and (**c**) *part-idx-merged*. The y-axis begins with chromosome 1 at 0 and ends with chromosome X, Y, and the mitochondria at the top. The length of rectangles along the x-axis are in the correct scale to the length of the read. However, the length along the y-axis are exaggerated to a fixed value so that it is clearly visible. In (**a** and **c**), the areas with dotted circles contain the differences between unique mappings for each alignment strategy. Circled regions in (**a**) map to a genomic locus harbouring the satellite repeat displayed in (**d**). Out of the 6 unique mappings in (**c**), the segment with the highest mapping quality (6) maps to the simple repeat containing region displayed in (**e**).

To evaluate how chimeric reads will be affected by aligning them to partitioned indexes, we tested this case on an ultra-long (473 kb) chromothriptic ONT read from a patient-derived liposarcoma cell line^20^. Chromothripsis is a genetic phenomenon often associated with cancer and congenital diseases. It is caused by several rounds of breakage-fusion-bridge, which produce complex and localised genomic rearrangements in a relatively short segment of DNA. The *single-idx* produced 41 (36 primary + 5 secondary mappings) mappings (Fig. 4a). However, *part-idx-no-merge* (16 partitions) produced 688 (608 primary + 80 secondary) mappings (Fig. 4b), while mapping with *part-idx-merged* resulted in 47 (42 primary + 5 secondary) mappings (Fig. 4c).

In *single-idx* and *part-idx-merged*, 34 mappings were the same. Interestingly, there were 7 mappings unique to *single-idx* and 6 unique to *part-idx-merged* (Supplementary Table S3). All 7 alignments unique to *single-idx* map to the centromeric region of chromosome 11 (Fig. 4d), which is composed of large arrays of repetitive DNA (also known as satellite DNA). The alignments that are unique to *part-idx-merged* map to simple repeats (e.g. GAGAGAGA).

### Memory usage and runtime of partitioned indexes

**Figure 5:**
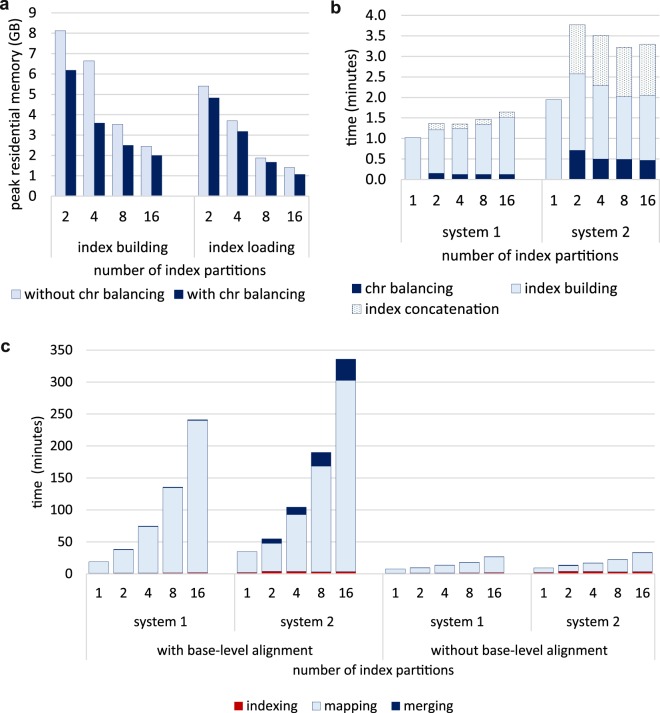
Peak memory usage and runtime for a partitioned index of the human genome. (**a**) Peak memory usage in function of the number of partitions for *ab initio* index generation (left) and loading a pre-built index (right). Dark bars represent memory usage when performing chromosome balancing as described herein, whilst light bars represent the default iterative partition distribution as implemented in Minimap2. (**b**) Detailed runtime metrics for index building across two computational systems. *System 1* is a laptop with flash memory (Intel i7-8750H processor, 16 GB of RAM and Toshiba XG5 series NVMe SSD) while *system 2* is a workstation with a mechanical hard disk (Intel i7-6700 processor, 16 GB of RAM and Toshiba DT01ACA series HDD). The total indexing time has been broken down into three steps; *chr balancing*, *index building* and *index concatenations*. *Chr balancing* includes the overhead for chromosome sorting, partitioning and writing each partition to a separate file. (**c**) Runtime for base-level alignment (left) and block/locus level mapping (right). System 1 and 2 are as described in (**b**). Alignment was performed on the NA12878 data (see Materials and methods) with the *map-ont* pre-set in Minimap2 using 8 threads. Runtime statistics are composed of *indexing* (index generation including the overhead), *mapping* (total time for aligning reads to each partition), and *merging* using the method described herein. Runtimes include file reading and writing.

In addition to comparable quality of alignments, using a partitioned index yields impressive reductions on peak memory usage during indexing. About 7.7 GB of memory is required to hold a single reference index, whereas only 1.5 GB is needed for a partitioned index with 16 parts (Fig. 5a). Peak memory usage can be further reduced by distributing or ‘balancing’ chromosomes across partitions based on their size (see Materials and methods). These indexing approaches combined with a mini-batch size between 5–20 Mbases (Minimap2 parameter *K*) enables alignment of long reads to the human genome with less than 2 GB of RAM. Although generating an index *ab initio* requires more memory than loading a pre-built one, this only needs to be done once for a given reference and can be performed *a priori*, if required.

The reduced peak memory usage of a partitioned index comes with an inherent sacrifice in computing speed. Alignment requires significantly more time than the balancing, indexing and merging steps when generating an index *ab initio*, which we observed to be relatively constant across different partition ranges (Fig. 5b). Less than 10% of the total compute time (5.7 h) for base-level alignment is dedicated to balancing, indexing and merging when mapping to 16 partitions with eight CPU threads and a mechanical hard drive. When using flash memory, the overheads have very minor impact (3% of the total compute time). Chromosome balancing, for instance, required less than 1 minute and merging required less than 2 minutes.

We observed that the alignment time increased less than linearly with the number of partitions in the index (Fig. 5c). Since our motivation is to reduce the memory requirements of mapping to large references, this will inevitably impact speed. However, this also facilitates parallelisation of alignments, where several small index partitions can be distributed across an array of low-memory devices (e.g. microcomputing boards such as Raspberry Pi). It also enables the use of mobile computing devices, such mobile phone or inexpensive laptops, which would otherwise be impossible. Considering that ONT’s MinION sequencer generates about 80% of all data in the first 24 h, using 16 partitions would enable real-time mapping on a system with 2 GB of RAM in parallel to data acquisition, whereas a system with 4 GB RAM would only require 4 partitions and less than 1 h of compute to align a typical ONT MinION dataset.

## Discussion

This work details two ways to reduce memory requirements for performing alignments on large genomes, or collections of genomes, using Minimap2. By tuning alignment parameters, peak memory usage can be lowered marginally, although with non-negligible impact to the accuracy of alignments. We demonstrated this effect by sequencing and mapping a diverse and representative set of synthetic spike-in controls, which can be used as a ground truth to assess sequencing and alignment accuracy. However, these data were not used to benchmark the alignment accuracy of Minimap2, but to demonstrate the comparative and relative impact of alignment parameters on memory usage. In this regard, we show that partitioning a large reference into smaller indexes upstream of an appropriate merging process drastically reduces the peak memory usage without compromising alignment accuracy.

Previous studies have described indexing strategies to improve computational efficiency. DIDA^15^ and DREAM-Yara^16^ use bloom filters to distribute sequencing reads to the most appropriate index partitions. However, these works employ methods that are dependent on indexes generated with the Burrows-Wheeler transform algorithm, which are ideal for short, less noisy reads generated by second generation sequencing platforms. These strategies focus on indexing enhancements centred on reducing the alignment search space by delivering reads to the most suitable index partition. Our work focuses on multi-index alignment merging, which is independent and complementary to these strategies. We reveal the problems derived from mapping to multiple partitions, such as the accuracy of mapping reads and the overestimation of the mapping quality. We show that our merging technique circumvents these issues via the analysis of mapping qualities and the use of independent controls. The partitioned reference approach has also been previously used for reducing the memory usage of BWA, a popular short read alignment program^17^. However, the final output consists of the indiscriminate concatenation of the alignments from all the partitions, raising several of the caveats exposed in Fig. 2. We have demonstrated that performing appropriate merging of alignment output is required to eliminate many mapping artefacts, thus improving overall accuracy.

We also showed that *part-idx-merged* can provide a better result than a simple strategy of filtering out results with low mapping qualities in *part-idx-no-merge*. This is supported by the results from synthetic reads, where the accuracy of alignments with mapping quality 60 in *part-idx-no-merge* is lower than those from *part-idx-merged*. Furthermore, a simple strategy to remove all short mappings from *part-idx-no-merge* is also less than ideal. In fact, *Paftools* (which was used for evaluating the synthetic read alignments) considers the longest primary mapping when multiple primary mappings exist to assess alignment accuracy.

However, *part-idx-merged* can sometimes generate non-identical alignments to that of *single-idx*. This is likely a consequence of slight variations in highly abundant k-mers observed during index construction. Overall, this affects only a few reads that would nonetheless have low mapping qualities–an issue that has previously been reported by the author and users of Minimap2 (see the public code repository associated with Li, H.^4^). Furthermore, the reported alignments might differ in long low-complexity regions, as Minimap2 may generate suboptimal alignments in long low-complexity regions (see Supplementary Materials of Li, H.^4^).

Although a partitioned index reduces peak memory usage, the runtime is proportionately higher. This is because all the reads should be repeatedly mapped to each partition of the reference. However, this strategy lends itself well to distributed computing, in particular when many smaller, less expensive computing devices are available.

A limitation of this method also lies in the maximal number of partitions an index can be split into, which currently depends on the longest chromosome or contig. We have not yet investigated the impact of splitting chromosomes into fragments, although we anticipate this would not drastically affect results (as exemplified from the chromothriptic read example above). Furthermore, we have not tested the impact of this strategy for RNA sequencing read alignment, which implements different alignment scoring metrics.

In addition to capability of mapping long reads to large genomes on devices with a small memory footprint, our extension to Minimap2 could potentially be useful for the following applications:

- *Mapping to huge reference genome databases*. Meta-genomic databases can be hundreds of gigabytes in size. Hence, holding the index for the whole database would be challenging even for high-specification servers. Especially when multiple species with similar genomes are present, an accurate mapping quality with correct flags, headers, and reduced output file sizes is always appreciated. Alternatively, mapping genome assembly contigs, or a select amount of long reads, to a large public sequence repository (akin to a BLASTN nucleotide databse query) could benefit from our approach. However, the effect of merging output from such large queries has yet to be investigated.
- *Mapping with a lower window size for increased sensitivity*. Minimap2 runs on a default minimiser window size of 10. However, reducing this value improves the mapping sensitivity, but increases the memory consumption. Our method can be beneficial for applications where high sensitivity may be preferred, for instance, when confronted to low coverage sequencing data.

While preparing this manuscript, our method was integrated into the source code of the original Minimap2 software repository. In Minimap2 version 2.12, the option--*split-prefix* can be used to align to a partitioned index. The developer of Minimap2 has expanded our implementation to support paired end short reads and multi-threading for the merging process. The original version we implemented for conducting the above experiments is available in the associated *github* repository^21^ and can be useful for understanding the underlying algorithm. The partitioned index functionality can be invoked with the option--*multi-prefix*. Instructions to run the tools are detailed in the Supplementary Notes.

## Materials and Methods

### Exploration of parameters that affect memory usage in Minimap2

For measuring peak memory usage and runtime, publicly available NA12878 ONT reads^14^ were aligned to the human genome reference (GRCh38) with Minimap2^4^. Peak memory usage and runtime were measured by using the GNU command line *time* utility with the *-v* option.

Sensitivity and error rate calculations for different window sizes (Minimap2 parameter *w*) were performed using Sequins, synthetic human genome spike-in controls and synthetic PacBio reads (see below). By reversing (not complementing) the sequences from regions of interest, these spike-in controls reproduce most features of the human genome, including nucleotide frequencies, somatic variation, low-complexity regions and repeats. Given their chiral or ‘mirror’ design, Sequins do not align to the native reference sequence but will align to a mirror copy of the human reference genome. They can thus be used to benchmark alignment accuracy when spiked-in to a normal sample, although they were sequenced in isolation for this study. The particular Sequins design we employed was unpublished at the time this manuscript was prepared (Deveson *et al*., under review), but it is conceptually similar to what is reported by Deveson *et al*.^18^. 1 *μ*g of Sequins DNA was sequenced on a ONT R9.4.1 flow cell, using the LSK108 sample preparation kit and the results were base called with ONT’s proprietary Albacore software (version 1.2.6). Reads were mapped to the reverse human genome, using Minimap2 under the preset *map-ont* for different window sizes.

We leveraged the chiral design of Sequins to qualify any mapping to the normal reference genome as a false positive. True positive Sequin alignments should display the exact mapping positions on the mirrored human genome, as intended by their design. However, given stochastic variations in sequencing (base calling idiosyncrasies, involuntary library fragmentation, sample degradation, etc) the primary mappings derived from the default window size parameter (*w* = 10) in Minimap2 were used as a reference to assess the relative effect and impact of parameter tuning. Then, for a given window size:

- *Mismatching mappings* refer to primary mappings that had different positions to the mappings with reference parameters;
- *Missing mappings* refer to primary mappings that were not observed in empirical alignments, but were observed in alignments with reference parameters;
- *Extra mappings* refer to primary mappings that were observed in empirical alignments, but were not observed in alignments with reference parameters.

The above counts were expressed as a percentage of the total number of reads. The sum of mismatch and extra mapping percentages was taken as an approximation of the relative error rate. The relative sensitivity was approximated by subtracting the percentage of missing mappings from 100.

### Merging of mappings from a partitioned index

We extended the partitioned index approach of Minimap2 to eliminate alignment artefacts as described below. The index partitioning in Minimap2 is inherited from the first version of Minimap^22^. This feature is for finding long read overlaps for use with assembly tools such as Miniasm^22^. As overlap computing requires all-vs-all mapping of reads, the index is built for chunks of 4 Gbases (can be overridden with the *-I* argument) at a time, effectively partitioning the alignment index to keep the maximum memory capped at around 27 GB. For each part of the index, Minimap attempts to map all the reads. The concatenated alignments from all the parts is the final output.

We modified Minimap2 to serialise and store the software’s internal state during the alignment process. The internal state is serialised in binary format to reduce disk usage. The internal state includes: (i) mapped positions, chaining scores and other mapping statistics for each alignment record; (ii) DP score, CIGAR string, and other base-level alignment statistics for each alignment record (when base-level alignment is specified); and (iii) sum of region length of read covered by highly repetitive k-mers for each read (referred to as repeat length). These data form the serialised binary files, one for each partition of the index.

When an alignment process has completed, we simultaneously open all the serialised binary files together with the queried sequence file. For each queried read (or contig), the previously serialised internal states of all alignments for the given read (resulting from all the index partitions) are loaded into memory. If no base-level alignment has been requested, the alignments are sorted based on the chaining score in descending order. Otherwise, the sorting is based on the DP alignment score in descending order. The classification of primary and secondary chains is reiterated as implemented in Minimap2. This corrects the primary and secondary flags in the output. Then, the secondary alignment entries are filtered based on a user requested number of secondary alignments, and the requested minimum primary to secondary score ratio, effectively removing spurious secondary alignments. If a SAM output has been requested, the best primary alignment is retained as the primary alignment and all other primary alignments are classified as supplementary alignments. An unaligned record is printed only if the read is not mapped to any part of the index.

The length of the read covered by repeat regions in the whole genome (repeat length) is one of the parameters required to estimate an ideal mapping quality (MAPQ). The MAPQ produced by Minimap2 is a globally computed heuristic that depends on a large number of parameters, including this repeat length. We estimate this global repeat length by taking the maximum of the previously serialised repeat lengths (for each partition of the index) for that particular read. The Spearman correlation between this estimated repeat length and the global repeat length is 0.9961. In theory, it would possible to exactly calculate this value by serialising and storing the positions of repeats within the read. However, as the MAPQ is itself an estimation and the accuracy of mappings was adequate in our initial tests, we simply took the maximum. Hence, the computed MAPQ during merging of a partitioned index is not exactly the same as for a single reference index, but very similar overall. This computed MAPQ is more accurate than a MAPQ computed only from the repeat length for a single part of the index.

Merging is performed in the order of input read sequences, and mappings for a particular read ID will be adjacent in the output. As the serialised data are loaded into memory for each read (or a batch of few reads) at a time, the memory usage of merging is only a few megabytes. For a detailed explanation of the merging algorithm refer to Supplementary Notes.

### Chromosome balancing

The construction of partitioned indexes in Minimap2 (specified by the *-I* option) processes reference sequences iteratively, which does not distribute reference sequences (i.e. chromosomes) evenly when using multiple partitions. We implemented a simple sorting and binning algorithm to mitigate this effect. First, a command line parameter describing the number of desired partitions is considered. Then, the reference sequences (or chromosomes) are sorted in descending order of size. Next, a chromosome is assigned to the bin (or partition) with the lowest sum of bases, and the sum of that bin is then incremented by the chromosome size. This effectively distributes the chromosomes to roughly balanced partitions in ${\mathscr{O}}$(*n* log *n*) time complexity–adding negligible overhead to the overall indexing process (Supplementary Table S4). We output the reference sequences belonging to the each bucket in a separate file. Finally we launch the Minimap2 indexer on each file and concatenate the indexes. This approach is available under *misc/idxtools* in the *github* repository^21^ and the instructions to run the tool are detailed in Supplementary Notes.

### Datasets and evaluation methodology

All experiments were performed using the human genome as a reference (GRCh38 with no ALT contigs). The scripts and tools written for performing the experiments are available under *misc/idxtools/eval* in the *github* repository^21^.

#### Synthetic reads

Mapping accuracy was evaluated using synthetic long reads. We generated about 4 million PacBio reads using PbSim^23^ under “Continuous Long Read” mode (long reads with a high error rate). The minimum, maximum and the mean read length were set to be 100 bases, 25 kbases and 3 kbases respectively with a standard deviation of 2300. The minimum, maximum and the mean accuracy of bases were set to 0.75, 1.00 and 0.78 respectively with a standard deviation of 0.02. The ratio between substitution:insertion:deletion was 10:60:30.

In the context of parameter tuning (Supplementary Fig. S2), the reads were mapped using Minimap2 with different window sizes while keeping other parameters constant. Then the accuracy evaluation was performed using the *Mapeval* utility in *Paftools*—part of the Minimap2 software package—where a read is considered correctly mapped if the mapping coordinates of its longest alignment overlap with the true reference coordinates with an overlap length of 10% or higher.

In the context of multi-part index accuracy, simulated long reads were aligned using Minimap2 with single reference index (*single-idx*), partitioned index without merging (*part-idx-no-merge*) and partitioned index with merging (*part-idx-merged*). Partitioned indexes with 2, 4, 8 and 16 parts were tested. For each instance, we evaluated base-level alignments (default SAM output) as well as locus- or block-based alignments (default PAF output without CIGAR information). To evaluate alignment accuracy, the *Mapeval* utility in *Paftools* was used with default options, which consider only the longest primary alignment for a read. However, *Paftools* assumes that all alignments for a particular read reside contiguously. Hence, for *part-idx-no-merge*, we first sorted the alignments based on the read ID. The output from *Paftools* contains the accumulative mapping error rate and the accumulative number of mapped reads for different mapping quality thresholds^24^. The fraction of mapped reads is taken as a measure of sensitivity.

#### Nanopore sequencing data

We could not find a suitable ONT read simulator. Published ONT read simulators explored at the time of writing had issues such as dependence on Minimap2 (would cause a bias), unavailability of trained models for the human genome, being unstable or unavailability of source code. For instance, DeepSimulator^25^ and NanoSim^26^ are dependent on Minimap2, SNaReSim^27^ code was not available. Hence, we used a a dataset from the publicly available NA12878 sample (rel3-nanopore-wgs-84868110-FAF01132^14^). The dataset had 689,781 reads totaling about 5.5 Gbases. We aligned this dataset to the human genome using a 16-part index with merging (*part-idx-merged*) and without merging (*part-idx-no-merge*) with base-level alignment. Then we compared those outputs by generating some alignment summary metrics with the result from a single reference index (*single-idx*). We initially attempted to perform an extensive comparison using tools such as the *CompareSAMs* utility in *Picard*^28^ and the *qProfiler* utility in *AdamaJava*^29^, however, they crashed probably because they are designed to work with short reads. Hence, we first obtained simple summary metrics using *samtools*^30^ together with custom Linux shell scripts. Then, we performed an extensive read by read comparison between the SAM outputs from *single-idx* and *part-idx-merged* using a custom tool written in C. The tool sequentially reads two SAM files while loading all the alignments for a particular read to the memory at a time. For a particular read, it compares and then outputs the alignment entries when the mapping positions between the two sets are disparate, or if they mapped only in one set (discordant). On these disparate and discordant mappings, we used *bedtools*^31^ to find overlaps with the UCSC Genome Browser repeatMasker track.

The above-mentioned NA12878 dataset was used to measure the runtime of partitioned indexes. The runtime and peak memory usage were measured using the GNU *time* command line utility.

The ultra-long chromothriptic read was sourced from an unpublished patient-derived dataset generated in house (see Garsed *et al*.^20^ for more details on the cancer cell line). The data was generated on a MinION MkI sequencer (MN16218) with MinKNOW version 1.1.17 on a first generation R9 flowcell (MIN105, no spot-on loading, flow cell ID FAD24075) using the SQK-RAD001 library preparation kit from ONT. The raw data for the read was live base called with MinKNOW 1.1.17 and produced an average fastq score of 7.8.

## Conclusion

Aligning long reads generated from third generation high-throughput sequencers to large reference genomes is possible on computers with limited volatile memory. Parameter optimisation alone cannot substantially reduce memory usage without considerably sacrificing alignment quality. Partitioning an alignment index, saving the internal state, and merging the output *a posteriori* substantially reduces memory usage. This strategy reduces the memory requirements for aligning ONT reads to the human reference genome from 11 GB to less than 2 GB, with minimal impact on accuracy.

## Supplementary information

Supplementary Information

## Data Availability

The datasets generated and analysed during the evaluation are available in the *figshare* repository^32^ [10.6084/m9.figshare.6964805.v1].
