# Supplementary material for: Featherweight long read alignment using partitioned reference indexes
Source: Sci Rep. 2019 Mar 13;9:4318. doi: 10.1038/s41598-019-40739-8 (PMC6416333; doi:10.1038/s41598-019-40739-8)
Supplement: Supplementary file 1 — Supplementary Information [file 41598_2019_40739_MOESM1_ESM.pdf]

# Supplementary materials - Featherweight long read alignment using partitioned reference indexes

**Hasindu Gamaarachchi[1,2], Sri Parameswaran[2], Martin A. Smith[1,3*]**

[1] Kinghorn Centre for Clinical Genomics, Garvan Institute of Medical Research, 370 Victoria St, Darlinghurst, NSW, Australia
[2] School of Computer Science and Engineering, UNSW Sydney, Kensington, NSW, Australia
[3] St-Vincent's Clinical School, Faculty of Medicine, UNSW Sydney, Darlinghurst, NSW, Australia.
* martinalexandersmith(at)gmail(dot)com

## List of Tables

S1 Memory usage of Minimap2 for default parameters . . . 2
S2 Statistics for alignment outputs for 689,781 reads from NA12878 . . . 2
S3 Mappings of the chromothriptic read which are different in *single-idx* and *16-part-idx-merged* . . . 2
S4 Detailed runtime for partitioned indexes . . . 3

## List of Figures

S1 Effect of the window size parameter on the MAPQ distribution for synthetic spike-in controls . . . 4
S2 Effect of the window size on the error rate and sensitivity for simulated reads . . . 4
S3 Features of alignments that were discordantly mapped in *single-idx* and *16-part-idx-merged* indexing strategies . 5
S4 Genome browser screenshots of alignments unique to the *16-part-idx-merged* that do not overlap annotated repeats 6
S5 Distribution of MAPQ and DP alignment score for the disparate mappings (different by at least one base position) between *single-idx* and *16-part-idx-merged* . . . 7
S6 Scatter plot of alignment scores of *single-idx* vs *16-part-idx-merged* for disparate alignments (different by at least one base position) . . . 7
S7 Distribution of genomic features of disparate alignments (different by at least one base position) between *single-idx* and *16-part-idx-merged* . . . 8
S8 Statistics and genomic features of disparate mappings (mapping positions not overlapping at least by 10%) between *single-idx* and *16-part-idx-merged* . . . 9

## Supplementary Notes

**Supplementary Note 1 - Detailed Methodology of the merging** **10**
**Supplementary Note 2 - Detailed Methodology of the chromosome balancing** **11**
**Supplementary Note 3 - Instructions to run the tools** **12**

### Table S1. Memory usage of Minimap2 for default parameters

| | PacBio | Oxford nanopore |
|---|---|---|
| **Index construction** | 8.67 GB | 11.32 GB |
| **Index residence** | 6.33 GB | 7.71 GB |
| **Mapping with base-level alignment (SAM output)** | 8.56 GB | 11.30 GB |
| **Mapping without base-level alignment (PAF output)** | 7.14 GB | 8.80 GB |

Minimap2 was run with default parameters. Pre-set profiles *map-pb* and *map-ont* were used for PacBio and Oxford Nanopore, respectively. The peak memory usage for each event is presented. Index construction refers to the building of the index and then serialising the index to a file. Index residence is the memory required only for the index to reside in memory, such as when loading a pre-built index.

### Table S2. Statistics for alignment outputs for 689,781 reads from NA12878

| | single-idx | 16-part-idx-no-merge | 16-part-idx-merged |
|---|---|---|---|
| **File size (SAM file)** | 12.1 GB | 180 GB | 12.4 GB |
| **No of SAM entries** | 862,427 | 23,749,310 | 969,223 |
| **No of unaligned entries** | 127,177 | 7,365,979 | 120,775 |
| **No of aligned entries** | 735,250 | 16,383,331 | 848,448 |
| **No of primary alignments** | 592,748 | 6,228,567 | 654,302 |
| **No of secondary alignments** | 142,502 | 10,154,764 | 194,146 |

### Table S3. Mappings of the chromothriptic read which are different in single-idx and 16-part-idx-merged

| Only in single-idx | | | Only in 16-part-idx-merged | | | | |
|---|---|---|---|---|---|---|---|
| | | | | | | | |
| **RefName** | **RefStart** | **RefEnd** | **ReadStart** | **ReadEnd** | **Strand** | **MAPQ** | **Type** |
| chr11 | 51616896 | 51619171 | 228106 | 230227 | + | 0 | Primary |
| chr11 | 51658200 | 51659147 | 229344 | 230227 | + | 0 | Secondary |
| chr11 | 51735238 | 51735692 | 229848 | 230263 | + | 0 | Secondary |
| chr11 | 51913079 | 51916719 | 226802 | 230227 | + | 0 | Secondary |
| chr11 | 53527640 | 53533417 | 226770 | 232447 | + | 0 | Primary |
| chr11 | 53696097 | 53697156 | 229848 | 230835 | + | 0 | Secondary |
| chr11 | 54005962 | 54006133 | 226668 | 226835 | + | 38 | Primary |
| chr5 | 61332327 | 61332440 | 156722 | 156833 | + | 0 | Primary |
| chr5 | 61332327 | 61332729 | 156586 | 156863 | + | 6 | Primary |
| chr6 | 125655477 | 125659733 | 371459 | 375714 | + | 0 | Primary |
| chr6 | 1610948 | 1611924 | 156014 | 156860 | + | 1 | Primary |
| chr8 | 60678764 | 60678812 | 156722 | 156770 | + | 0 | Secondary |
| chr8 | 60678764 | 60678812 | 156806 | 156860 | + | 0 | Secondary |

The alignments which were only found in the *single-idx* mapped to locations in the range chr11:51616896-54006133. The ones unique to *16-part-idx-merged* mapped to repetitive regions in chr5, chr6 and chr8. chr5:61332327-61332729, chr6:1610948-1611924 and chr8:60678764-60678812 contained simple repeats. chr6:125,655,477-125,659,733 had simple repeats,SINE repeats and LTR repeats

**Table S4.** **Detailed runtime for partitioned indexes**

| | | system 1 | | | | | system 2 | | | | |
|---|---|---|---|---|---|---|---|---|---|---|---|
| | **number of partitions** | **1** | **2** | **4** | **8** | **16** | **1** | **2** | **4** | **8** | **16** |
| **Indexing** | chr balancing (min) | 0.00 | 0.15 | 0.12 | 0.12 | 0.13 | 0.00 | 0.71 | 0.50 | 0.49 | 0.47 |
| | index building (min) | 1.02 | 1.06 | 1.11 | 1.21 | 1.38 | 1.95 | 1.87 | 1.79 | 1.53 | 1.58 |
| | index concatenation (min) | 0.00 | 0.16 | 0.12 | 0.13 | 0.14 | 0.00 | 1.20 | 1.22 | 1.20 | 1.25 |
| | **total indexing (min)** | **1.02** | **1.37** | **1.35** | **1.47** | **1.64** | **1.95** | **3.77** | **3.51** | **3.22** | **3.29** |
| **mapping with base-level alignment** | index loading (min) | 0.23 | 0.19 | 0.24 | 0.27 | 0.28 | 1.51 | 1.48 | 1.58 | 1.44 | 1.47 |
| | mapping (min) | 17.86 | 36.03 | 72.57 | 133.38 | 238.33 | 32.75 | 43.72 | 89.01 | 165.29 | 299.14 |
| | merging (min) | 0.00 | 0.88 | 0.90 | 0.93 | 1.07 | 0.00 | 7.27 | 11.69 | 21.39 | 33.43 |
| | **total mapping (min)** | **18.10** | **37.10** | **73.71** | **134.58** | **239.68** | **34.27** | **52.46** | **102.28** | **188.12** | **334.05** |
| **mapping without base-level alignment** | index loading (min) | 0.18 | 0.19 | 0.24 | 0.26 | 0.26 | 1.17 | 1.28 | 1.41 | 1.40 | 1.45 |
| | mapping (min) | 6.54 | 7.84 | 11.82 | 16.57 | 24.93 | 7.16 | 9.03 | 13.29 | 18.68 | 29.63 |
| | merging (min) | 0.00 | 0.27 | 0.27 | 0.25 | 0.27 | 0.00 | 0.63 | 0.23 | 0.34 | 0.61 |
| | **total mapping (min)** | **6.73** | **8.31** | **12.33** | **17.08** | **25.46** | **8.33** | **10.95** | **14.92** | **20.42** | **31.69** |

*System 1* is a laptop with flash memory (Intel i7-8750H processor, 16GB of RAM and Toshiba XG5 series NVMe SSD) while *system 2* is a workstation with a mechanical hard disk (Intel i7-6700 processor, 16GB of RAM and Toshiba DT01ACA series HDD). Alignment was performed on the NA12878 Nanopore data with the *map-ont* pre-set in Minimap2 using 8 threads.

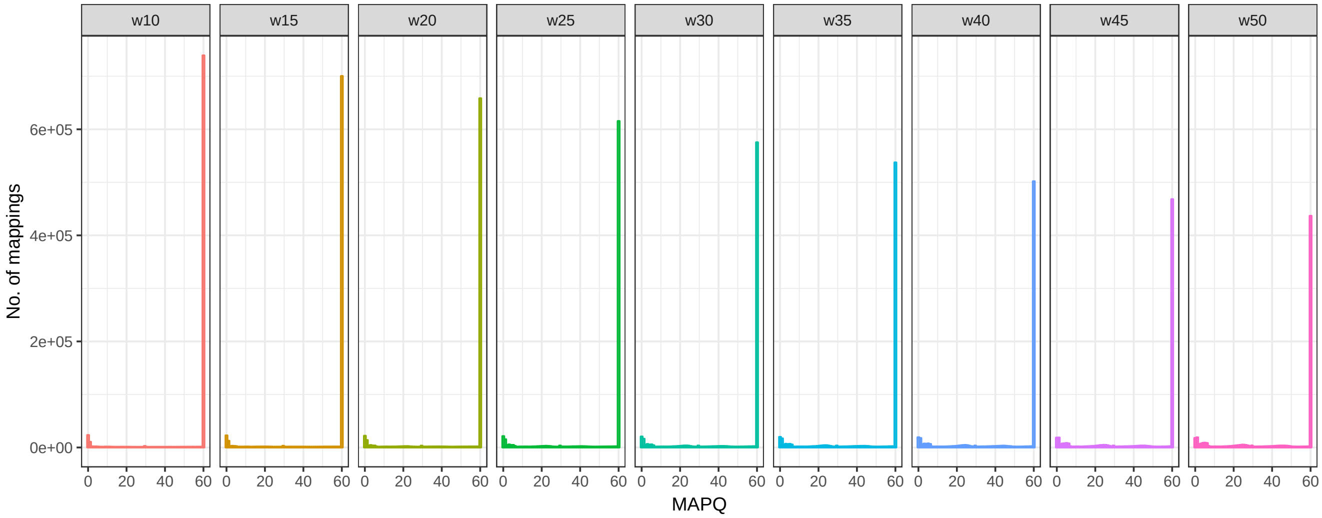


**Figure S1. Effect of the window size parameter *w* on the distribution of mapping qualities (MAPQ) for synthetic spike-in controls.**

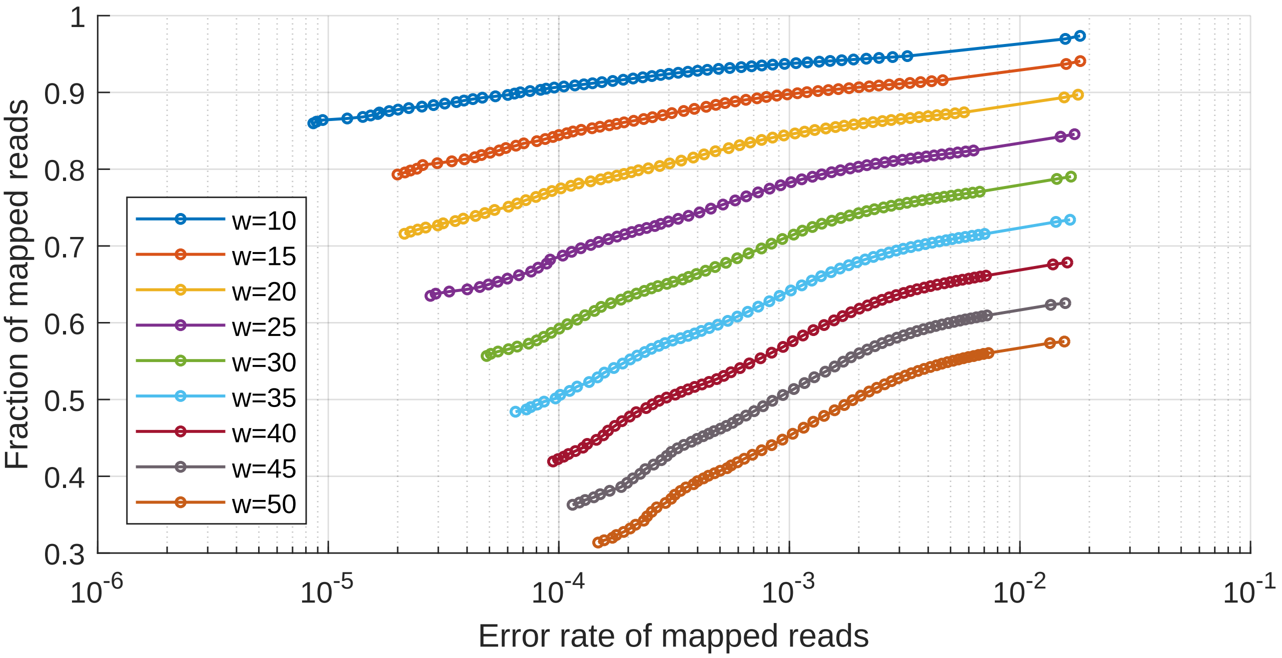


**Figure S2. Effect of the window size parameter *w* on the error rate and sensitivity for simulated reads.** Effect of window size on the proportion of mapped reads and the associated error rate (log scale) for 4 million simulated long reads (see Materials and Methods). A single curve contains points for each mapping quality threshold (MAPQ score), one point for each mapping quality threshold from 60 (leftmost) to 0 (rightmost).

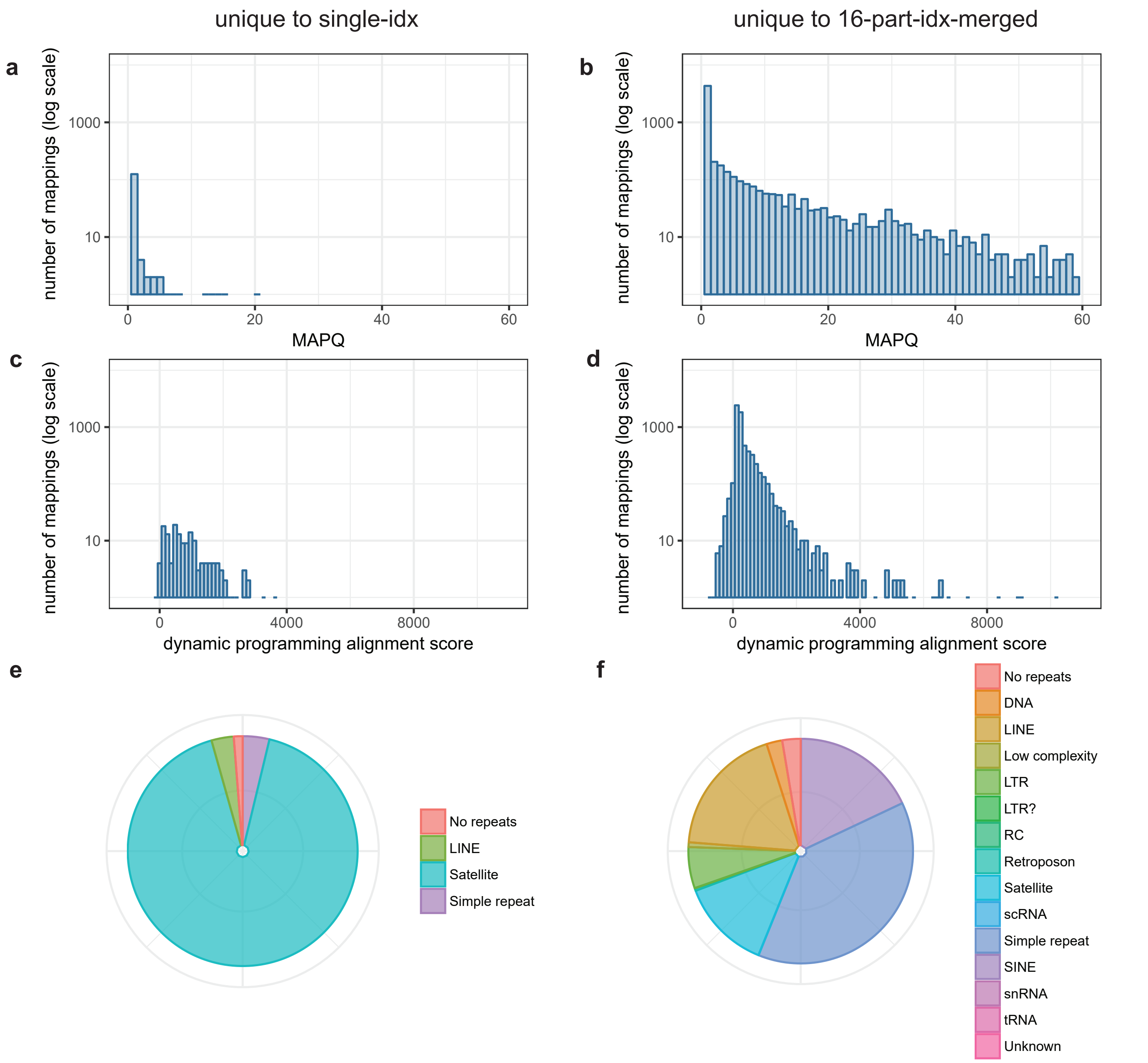


**Figure S3.** **Features of alignments that were uniquely mapped in single (left column) and multiple (right column) partition indexing strategies.**
**(a)** and **(b)** are the distribution of mapping quality scores (MAPQ). **(c)** and **(d)** are the distribution of dynamic programming alignment scores. **(e)** and **(f)** are the proportion of reads that map to regions of the human genome annotated as repeat elements (Repeat masker track of GRCh38 UCSC genome browser).

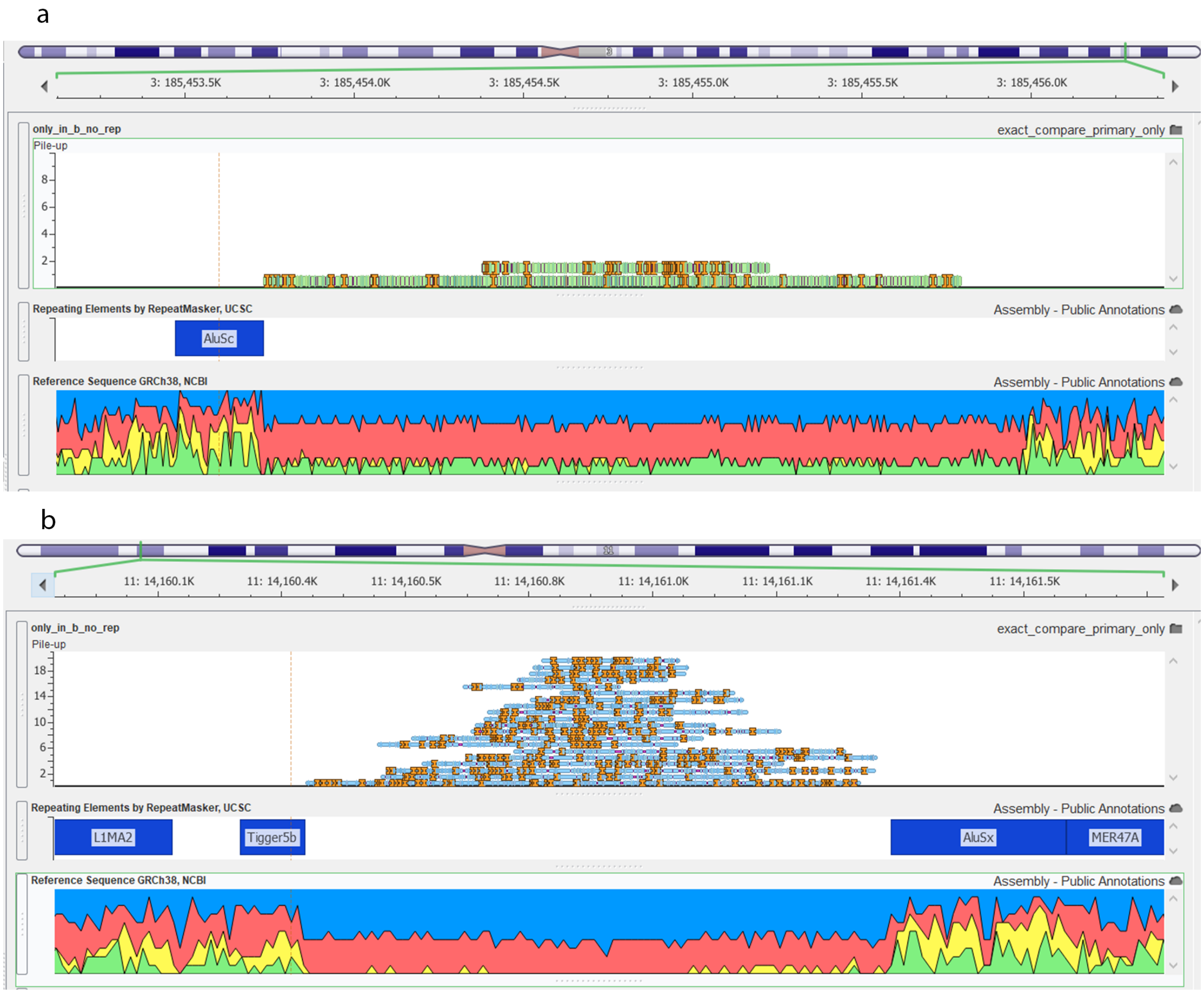


**Figure S4. Genome browser screenshots of alignments unique to the 16-part index that do not overlap annotated repeats.**
Out of the 281 alignments only found in *16-part-idx-merged* with no overlap with repeat regions, the two highest scoring alignments were found in the regions (a) chr3:185,453,170-185,456,442 and (b) chr11:14,160,389-14,161,287 respectively. The screen shots are from the Golden Helix GenomeBrowse [http://goldenhelix.com/products/GenomeBrowse]. The first track of each screen shot shows the pile-up of the alignments for the genomic region. The second track shows the repeat elements from the UCSC Repeatmasker track for GRCh38. The third track visualises the relative sequence composition of the GRCh38 reference for the particular region (A - red, C - yellow, G - green and T - blue).

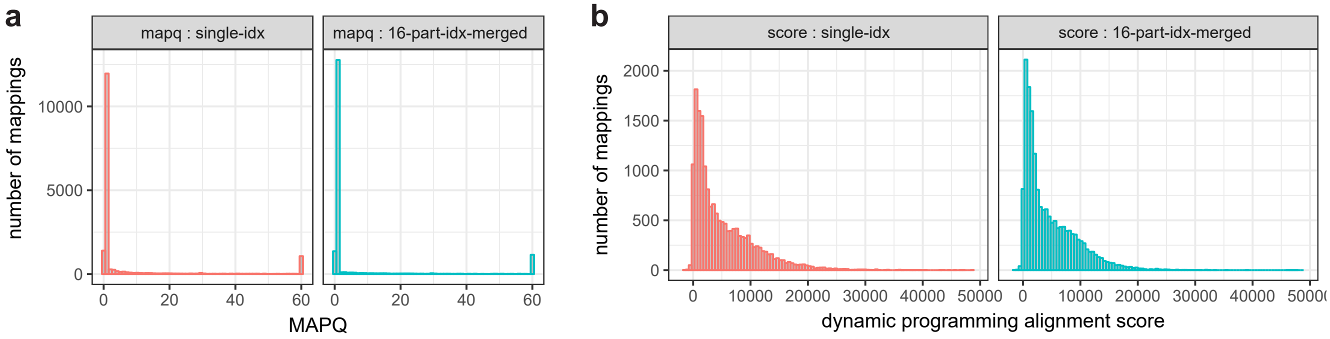


**Figure S5. Distribution of (a) mapping quality and (b) dynamic programming alignment score for the disparate mappings (different by at least one base position) between *single-idx* (left) and *16-part-idx-merged (right)*.**
Disparate mappings here refer to the 17,146 aligned NA12878 reads with different primary mappings (different by at least one base position) between the two partition strategies.

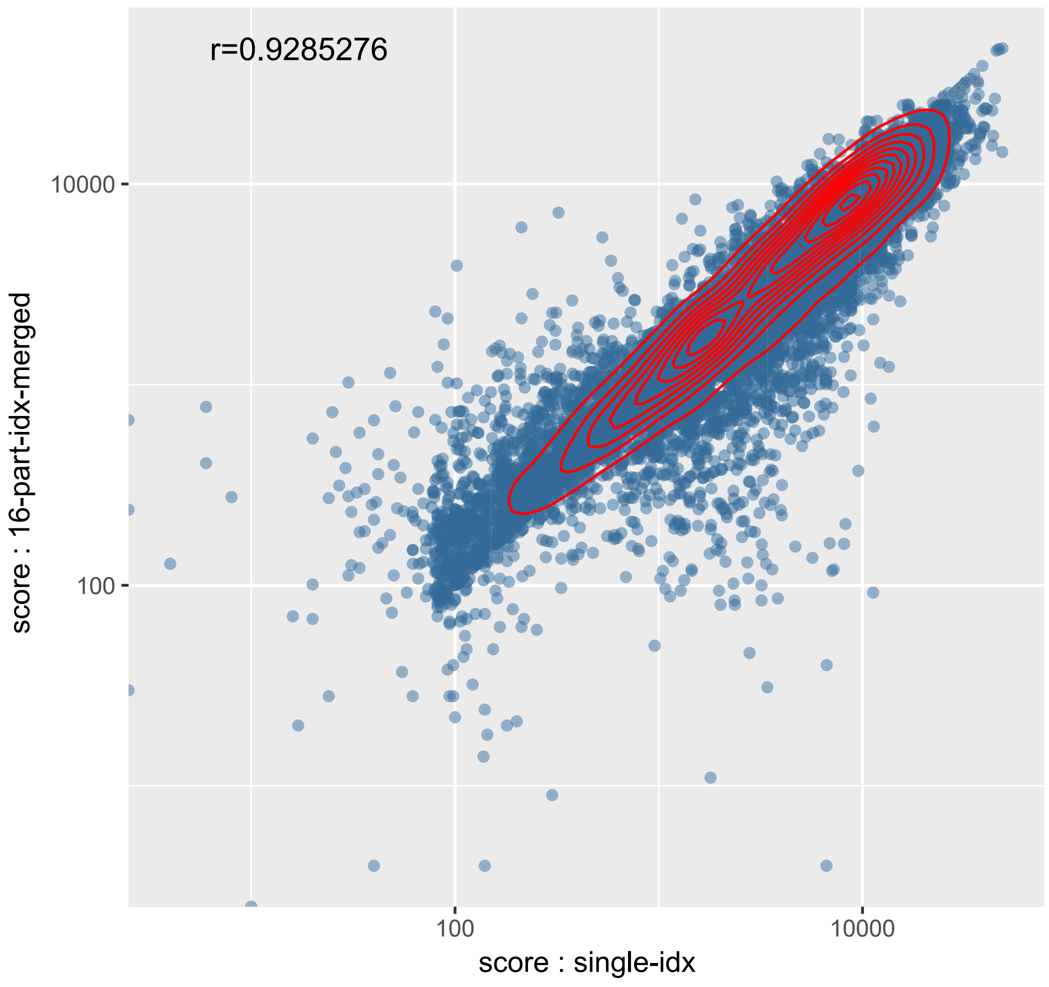


**Figure S6. Scatter plot of alignment scores of *single-idx* vs *16-part-idx-merged* for disparate alignments (different by at least one base position).**
The scatter plot contains 17,146 points representing each disparate mapping - different by at least one base position (Pearson's correlation (r) of 0.9285). The x and y axes are in log scale. 50.5% had higher dynamic programming alignment scores for *single-idx*, while 42.9% had higher scores in the *16-part-idx-merged*.

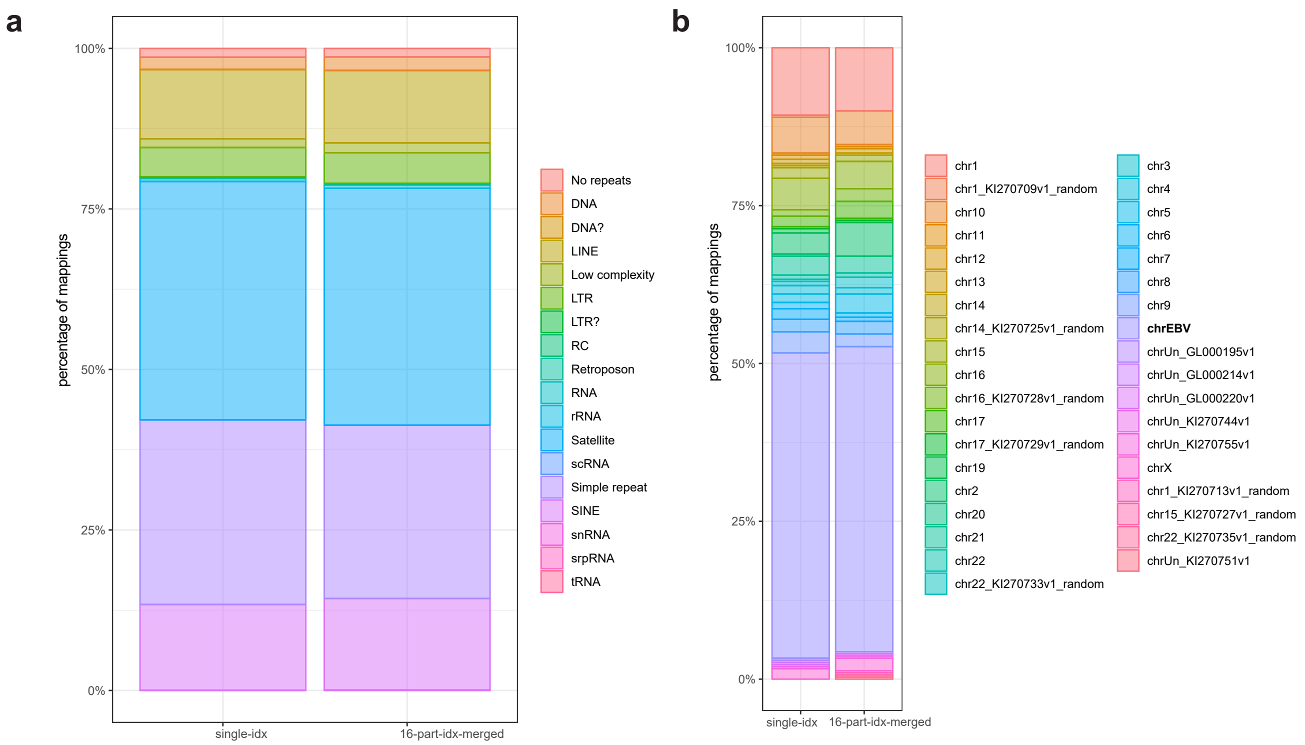


**Figure S7. Distribution of genomic features of disparate alignments (different by at least one base position).** (**a**) The distribution of repeat diversity in the disparate alignments (different by at least one base position) between *single-idx* and *16-part-idx-merged*. (**b**) Distribution of genomic targets for the 456 (left) and 472 (right) disparate alignments (that did not overlap with annotated repeat regions) between *single-idx* and *16-part-idx-merged*, respectively.

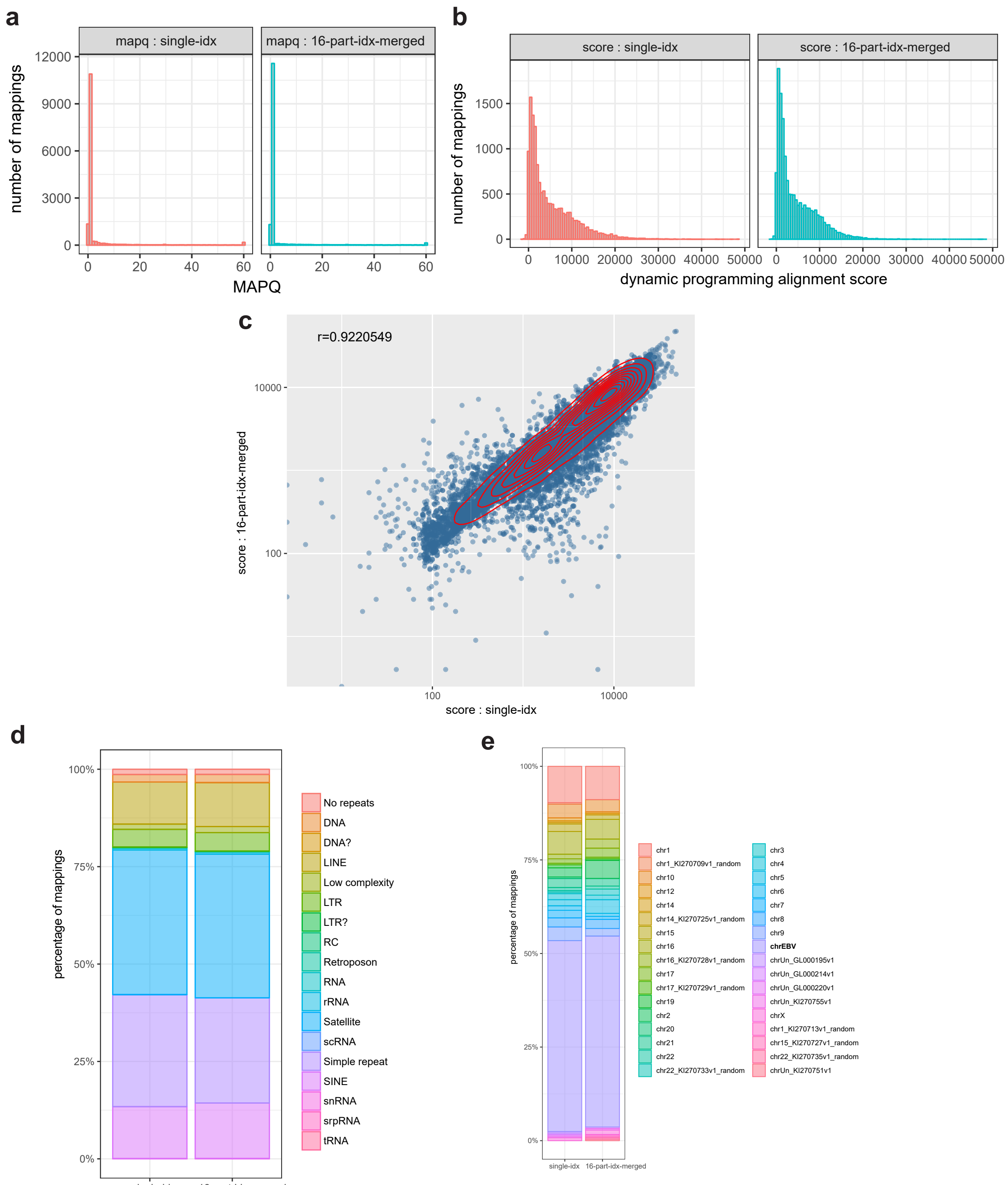


**Figure S8.** **Statistics and genomic features of disparate mappings (mapping positions not overlapping at least by 10%) between *single-idx* and *16-part-idx-merged***

Out of the 17,146 mappings that were different by at least one base position, 14,398 did not even overlap by 10% or more with their mapped locations on the reference. For those 14,398 mappings the distribution of the *(a)* mapping qualities and *(b)* alignment scores, *(c)* the scatter plot between the alignment scores, *(d)* the distribution of repeat diversity and *(e)* the mapping location of the mappings that did not contain repeats were almost identical to respective plots for disparate alignments –different by at least one base position.

## Supplementary Note 1 - Detailed Methodology of the merging

This supplementary note elaborates the merging method in detail together with some implementation details.

### Serialising (dumping) of the internal state

For each part of the partitioned index, a separate intermediate file (which we refer to as a dump) is created in the binary format [refer line 36-44 in https://github.com/hasindu2008/minimap2-arm/blob/v0.1-alpha/merge.c]. After a read is aligned to the partition of the index currently in memory, all the intermediate states for its alignments are dumped into this binary file [line 501-506 in https://github.com/hasindu2008/minimap2-arm/blob/v0.1-alpha/map.c]. Binary format was preferred as it reduces the file size compared to ASCII. When the last read is mapped to the current partition of the index in memory, the dump will contain the intermediate state of the mappings for all the reads, in the same order as the reads in the input read set. If the partitioned index had n partitions, at the end of the $n^{th}$ partitions we will have n such dumps.

The dumped internal state includes; fifteen 32-bit unsigned integers (such as the reference ID, chaining scores, query and reference start and end), two 32-bit signed integers and one floating point value. All these information are inside a single structure in Minimap2 (called *mm_reg1_t* in *minimap.h*) which made the dumping convenient. The size required for a single alignment is around 80 bytes.

If the user has requested Minimap2 to generate the base-level alignment, then the internal state for base-level alignment are also dumped. Base-level alignment information include; six 32-bit integers (such as the base level alignment score, number of CIGAR operations and a variable size flexible integer array for storing CIGAR operations. These information are stored inside another structure in Minimap2 (called *mm_extra_t*), which is only allocated if the base level alignment has been requested. The memory address to this structure is stored as a pointer in the previously mentioned *mm_reg1_t* structure. When dumping, we flatten the information linearly (eliminate memory pointers) to the file.

In addition to the above, a quantity called *replen* (sum of lengths of regions in the read that are covered by highly repetitive k-mers) is dumped. This is a per read quantity. We save the *replen* to the same dump file that we discussed above, just after the information for each mapping. For each read there will be a *replen* for each part of the index, that is saved in the dump for that particular part of the partitioned index [line 495 of https://github.com/hasindu2008/minimap2-arm/blob/v0.1-alpha/map.c].

### Merging operation

When alignment of all reads to all parts of the index completes, the merging operation is invoked [*merge* function in https://github.com/hasindu2008/minimap2-arm/blob/v0.1-alpha/merge.c]. We simultaneous open the read file and the dump files for all parts of the partitioned index. Reads are sequentially loaded while loading all the internal states for the alignments of that read. This includes the internal state for all its alignments (includes the base-level information if it had been requested) as well as the *replen* from each dump file. The flattened data in the files are restored to their original structures when loading to the memory.

If no base-level alignments had been requested, the alignments are sorted based on the chaining score in descending order [function *mm_hit_sort_by_score* in https://github.com/hasindu2008/minimap2-arm/blob/v0.1-alpha/merge.c]. If base-level alignment had been requested, they are sorted based on the base-level DP alignment score. Categorisation of primary and secondary chains is performed on the sorted alignments according to the same method done on Minimap2 (using *mm_set_parent* function). This fixes the issue with the primary vs secondary flag. Then the alignment entries are filtered based on the user requested number of secondary alignments and the priority ratio (using *mm_select_sub* function). This eliminates the issue of outputting secondary alignments for each part of the index that makes the output size huge. If the output has been requested in form of a SAM file, the best primary alignment is set to the primary flag while all other primary alignments are set to supplementary (using *mm_set_sam_pri* function).

The mapping quality (MAPQ) estimation depends on the length of the read covered by repeat regions in the genome. To compute a perfect value for this quantity, the whole index needs to be in the memory which is the case for a single reference index. However, we estimate this quantity by taking the maximum out of the *replen* values that were dumped for the particular read. The Spearman correlation of this estimated value to the perfect *replen* was 0.9961. As the mapping quality is anyway an estimation, computing the mapping quality based on the estimated *replen* does not affect the final results significantly.

### Emulated single reference index

For memory efficiency, Minimap2 stores meta-data of reference sequences (such as the sequence name and sequence length) only in the reference index (refer to *mm_idx_t* struct in *minimap.h*). The order in which the sequences reside in the *struct* array forms a unique numeric identifier for each reference sequence.

In the internal state for mappings only this numeric identifier is stored. The meta-data for the reference sequence are resolved using these numeric identifiers, only during the output printing. However, during merging we do not have the reference indexes in memory and the numeric identifiers cannot be resolved. Hence, we construct an emulated single reference index. For this, we save the meta-data of the reference sequences when each part of the partitioned index is loaded [line 47-54 in https://github.com/hasindu2008/minimap2-arm/blob/v0.1-alpha/merge.c]. These meta-data go to the beginning of the dump file for the particular part of the index. At the beginning of the merging, the meta-data is loaded back to form an emulated single reference index [line 164-173 in https://github.com/hasindu2008/minimap2-arm/blob/v0.1-alpha/merge.c]. However, the numeric identifiers in the internal states from the dump files are incorrect (as numeric the identifier is an independent incrementing index for each part of the index). These are corrected to be compatible with the numeric identifiers in the emulated single reference index by adding the correct offset [line 254 in https://github.com/hasindu2008/minimap2-arm/blob/v0.1-alpha/merge.c].

As a side effect of this emulated single reference index, a correct SAM header can be output even in the partitioned mode. Further, the merging process which merges the mappings for a read at a time, outputs the mappings for a particular read ID adjacently. Hence, no additional sorting is required for any downstream analysis tools that require so.

## Supplementary Note 2 - Detailed Methodology of the chromosome balancing

### Memory efficiency for references with unbalanced lengths

The existing partitioned index construction method in Minimap2, does not balance the size of index partitions when the reference genome has sequences (chromosomes) with highly varying lengths. This existing index construction method puts the reference sequences to the index in the order they exist in the reference genome. When constructing a partitioned index, it moves to the next part of of the index only when the user specified number of bases per index (by default 4 Gbases) is exceeded. When building a partitioned index for overlap finding, the parts would be approximately equal in size as the length of the longest read would be a few mega bases. However, in case of a reference genomes like the human genome where the chromosomes are of highly variable lengths, the size of the parts are unbalanced. The largest part of the index determines the peak memory. Hence, an unbalance will hinder the maximum efficiency for systems with limited memory. For instance, consider a hypothetical genome (total length 700M) with following chromosomes and lengths in the order chr1 (300M), chr2 (320M), chr3 (60M), chr4 (20M). Providing a value of 350M as the number of bases in a partition (with the intention of splitting into 2 parts), will create an unbalanced index as follows.

- part1 : chr1, chr2 : total length - 620M
- part2 : chr3, chr4 : total length - 80M

We follow a simple partitioning approach to balance this out. Instead of the number of bases per partition, the number of partitions is taken as a user input. The reference sequences are first sorted in descending order based on the sequence length (length without the ambiguous N bases). The sum of bases in each partition is initialised to 0. The, the sorted list in traversed in order while assigning the current sequence into the partition with the minimum sum of bases. The sum of bases in that partition is updated accordingly. Using this strategy, we get a distribution as follows.

- part1 : 300M, 60M : total length - 360M
- part2 : 320M, 20M : total length - 340M

## Supplementary Note 3 - Instructions to run the tools

### Example

1. Download and compile minimap2 that supports partitioned indexes and merging

```
wget https://github.com/hasindu2008/minimap2-arm/archive/v0.1.tar.gz
tar xvf v0.1.tar.gz && cd minimap2-arm-0.1 && make
```

2. Download the human reference genome and create a partitioned index with 4 partitions

```
wget -O hg38noAlt.fa.gz http://bit.ly/hg38noAlt && gunzip hg38noAlt.fa.gz
./misc/idxtools/divide_and_index.sh hg38noAlt.fa 4 hg38noAlt.idx ./minimap2 map-ont
```

   Note : http://bit.ly/hg38noAlt redirects to
   ftp://ftp.ncbi.nlm.nih.gov/genomes/all/GCA/000/001/405/GCA_000001405.15_GRCh38/seqs_for_alignment_pipelines.ucsc_ids/GCA_000001405.15_GRCh38_no_alt_analysis_set.fna.gz

3. Download a Nanopore NA12878 dataset and run Minimap2 with merging

```
wget -O na12878.fq.gz http://bit.ly/NA12878
./minimap2 -a -x map-ont hg38noAlt.idx na12878.fq.gz --multi-prefix tmp > out.sam
```

   Note : http://bit.ly/NA12878 redirects to
   http://s3.amazonaws.com/nanopore-human-wgs/rel3-nanopore-wgs-84868110-FAF01132.fastq.gz

Notes :

- To perform mapping without base-level alignment use:

```
./minimap2 -x map-ont hg38noAlt.idx na12878.fq.gz --multi-prefix tmp > out.paf
```

- From Minimap2 version 2.12-r827 [https://github.com/lh3/minimap2/blob/master/NEWS.md#release-212-r827-6-august-2018] onwards, the merging functionality has been integrated into the main repository. This version additionally supports paired-end short reads and the merging operation is multi-threaded. Use `--split-prefix` option instead of `--multi-prefix`.

### Index construction with chromosome size balancing

`divide_and_index.sh` is the wrapper script for balanced index construction. It takes the reference genome and outputs a partitioned index optimised for reduced peak memory. Its usage is as follows:

```
usage : ./divide_and_index.sh <reference.fa> <num_parts> <out.idx> <minimap2_exe>
<minimap2_profile>

reference.fa - path to the fasta file containing the reference genome
num_parts - number of partitions in the index
out.idx - path to the file to which the index should be dumped
minimap2_exe - path to the minimap2 executable
minimap2_profile - minimap2 pre-set for indexing (map-pb or map-ont)

Example : ./divide_and_index.sh hg19.fa 4 hg19.idx minimap2 map-ont
```

Functionality of `divide_and_index.sh` is as follows.

1. Compiling `divide.c` using `gcc` to produce `divide`.
2. Calling the compiled binary `divide` to split the reference genome into partitions such that the total length of chromosomes in each partition are approximately equal.
3. Calling the minimap2 binary separately on each reference partition to produce a separate index file for each partition.
4. Combining all the index files to produce a single partitioned index file.

### Running Minimap2 on a partitioned index with merging

To run minimap2 on an index created using the above method :

```
minimap2 -x <profile> <partioned_index.idx> <reads.fastq> --multi-prefix <tmp-prefix>
```

`--multi-prefix` which takes a prefix for temporary files, enables the merging of the outputs generated through iterative mapping to index partitions.
